# “Molecular Profiling and Ways Towards Personalized Medicine in Advanced Differentiated Thyroid Cancer”

**DOI:** 10.2174/138920291503140609094751

**Published:** 2014-06

**Authors:** Alessandro Antonelli

 The increasing incidence of thyroid nodules and cancer is associated with a higher demand for the diagnosis of thyroid nod-ules, and the treatment of this aggressive disease.

Molecular biology in the last decades has given important contributions in the understanding of the pathogenesis of thyroid cancer, and it is successfully used for the diagnosis of thyroid nodules. An important diagnostic impact can be achieved by testing fine needle aspiration samples, from thyroid nodules, for a panel of mutations that typically includes BRAF, RAS, RET/PTC, and PAX8/PPARg. The use of these and other emerging molecular markers is expected to improve significantly the accuracy of cancer diagnosis in thyroid nodules, overall in those with undeterminate cytology (Thyr 3). With this aim many researches evaluated (by new promising techniques) proteomics, and metabolomics of fine needle aspiration.

Mortality rates from thyroid cancer mainly depend on its aggressiveness. Geno- and pheno-typing of aggressive thyroid cancer has advanced our understanding of treatment failures and of potential future therapies. Unraveling molecular signaling pathways of thyroid cancer including its aggressive forms will hopefully pave the road to reduce mortality but also morbidity from this cancer.

Reliable pre-operative identification of high-risk patients may productively guide the initial surgical management since re-operative neck surgery is associated with increased morbidity. Pre-operative knowledge of mutations typically associated with thyroid cancer could alter the initial surgical treatment for at least 20% of patients and can potentially prevent the increased morbidity associated with reoperative neck exploration.

Despite the generally good prognosis of differentiated thyroid carcinoma (DTC), about 5% of patients will develop metastases which fail to respond to radioactive iodine (RaI), and other traditional therapies, exhibiting a more aggressive behavior. The advent of low-cost individual genomic analysis provides hope that we are entering a new era of personalized, patient-specific care. The recent advances in the pathogenesis of these diseases have revealed key targets (BRAF, RAS, RET, VEGFR, EGFR, etc.) that are now being evaluated in the clinical setting. The lack of effective therapies for DTC, resistant to radioiodine and traditional therapies, is now being overcome by the development of targeted novel compounds that have been demonstrated to induce clinical responses and stabilization of the disease. Interestingly, the most promising responses have been reported in patients treated with antiangiogenic inhibitors such as vandetanib and XL184 in medullary thyroid cancer, and sorafenib in papillary and follicular DTC.

However, the increasing complexity of the diagnostic and therapeutic tools for thyroid cancer needs, more and more, effort to personalize the diagnosis and the treatment, to reach the maximum success, avoiding unnecessary and potentially harmful treatments.

The aim of this Mini-Thematic Issue was to present and to discuss the most recent advancements to personalize the diagnosis and the treatment of thyroid nodules and cancer.

